# Association of sugar intake with incident dementia in the UK Biobank: a prospective cohort study

**DOI:** 10.1016/j.tjpad.2025.100311

**Published:** 2025-08-05

**Authors:** Yue Che, Wenming Wei, Tingting Mao, Lina Qin, Hanchi Wang, Yijia Li, Weixuan Da, Jin Feng, Li Liu, Bolun Cheng, Huan Liu, Yan Wen, Yumeng Jia, Feng Zhang

**Affiliations:** a NHC Key Laboratory of Environment and Endemic Diseases, School of Public Health, Health Science Center, Xi'an Jiaotong University, Xi’an, Shaanxi, 710061, China; b Collaborative Innovation Center of Endemic Diseases and Health Promotion in Silk Road Region, Xi’an, Shaanxi, 710061, China

**Keywords:** Dementia, Sugar intake, Sugar subtypes, Sex differences, Cox proportional hazards model, UK Biobank

## Abstract

**Background:**

Excessive sugar intake has been implicated in increased dementia risk; however, existing studies are constrained by small sample sizes and a primary focus on total sugar, with limited investigation into specific sugar subtypes. This study explores the relationship between sugar intake, its subtypes, and the incidence of dementia.

**Methods:**

We analyzed 172,516 participants from the UK Biobank who completed at least one 24-hour dietary recall (Oxford WebQ). Cox proportional hazards models estimated the hazard ratios (HRs) and 95 % confidence intervals (95 % CIs) for total sugar and its subtypes (free sugar, fructose, glucose, sucrose, maltose, lactose, and other sugars) about the risk of dementia. Sex-stratified analyses were also performed.

**Results:**

Higher intakes of total sugar (HR = 1.292, 95 % CI = 1.148–-1.453) and free sugar intake (HR = 1.254, 95 % CI = 1.117–-1.408) were significantly associated with increased dementia risk. Positive associations were also observed for non-milk extrinsic sugars (HR = 1.321, 95 % CI = 1.175–-1.486) and sucrose (HR = 1.291, 95 % CI = 1.147–-1.452). These associations were evident in women, with higher intakes of total sugars, free sugars, glucose, sucrose, and non-milk extrinsic sugars independently linked to increased dementia risk, whereas no significant associations were found in men.

**Conclusion:**

Higher consumption of total sugars, free sugars, sucrose, and non-milk extrinsic sugars confers increased dementia risk, particularly among women.

## 1 Introduction

Dementia is a clinical syndrome that primarily affects cognitive functions, including attention, memory, language, executive function, and processing speed [1]. Given the global acceleration of population aging, the prevalence of dementia is expected to rise significantly. The number of people affected by dementia worldwide is projected to increase from 57.4 million in 2019 to more than 150 million by 2050, representing a 166 % increase. Dementia has become one of the major threats to the health and quality of life of older individuals [2]. Although recent advances in the early treatment of dementia, particularly those targeting amyloid-associated lesions, have provided hope for patients, preventive measures aimed at delaying the onset of dementia remain a critical and cost-effective approach [3].

Sugar, classified into monosaccharides, disaccharides, and polysaccharides based on its chemical structure, is the body’s primary source of energy [4]. The brain, being one of the most energy-demanding organs, relies almost exclusively on glucose to fuel its complex physiological functions [5]. Excessive sugar consumption has been linked to cardiovascular diseases, metabolic disorders, and systemic inflammation [6].

Recent epidemiological studies have found that sugar intake may be closely associated with the development of dementia [7]. Excessive sugar consumption damages neurons in the brain, impairs cognitive function, increases the risk of dementia, and may contribute to cerebrovascular diseases [8]. Additionally, excessive sugar intake is linked to chronic conditions such as diabetes and obesity, which are known risk factors for dementia [[9], [10], [11]]. Based on these findings, we hypothesize that excessive sugar intake and its subtypes may increase the risk of dementia compared to low sugar intake.

However, significant controversies and limitations remain in the current research on the relationship between sugar intake and the risk of dementia [12]. Most previous studies were based on small sample sizes and primarily focused on a single type of sugar, overlooking the complexity of sugar’s presence in food as multiple subtypes. This narrow focus has led to certain limitations and uncertainties in the study results [13,14].

In this study, we examined the relationship between sugar intake and its subtypes with the incidence of dementia using a large dataset from the UK Biobank. This provides new insights into the treatment and prevention of dementia.

## 2 Methods

### 2.1 Study design and study population

The UK Biobank (UKB) is a large-scale, population-based cohort study that recruited over 500,000 participants aged 37 to 73 years between 2006 and 2010 in the United Kingdom (http://www.ukbiobank.ac.uk/about-biobank-uk/). The following exclusion criteria were applied in this study: (1) participants who did not complete at least one 24-hour dietary recall; (2) participants who reported implausible energy intakes (<500 or >3,500 kcal/day for women; <800 or >4,000 kcal/day for men); (3) individuals with missing data on covariates; and (4) participants diagnosed with dementia at baseline.

After data cleaning and screening, a total of 172,516 eligible participants were included in the final analysis. The UK Biobank received ethical approval from the National Information Governance Board for Health and Social Care and the National Health Service (NHS) North West Centre for Research Ethics Committee (Ref: 11/NW/0382). All participants provided informed consent via electronic signature.

### 2.2 Sugar intake measurement

Dietary data in the UK Biobank were collected using the Oxford WebQ, a web-based questionnaire designed to assess participants’ dietary intake over the previous 24 h. For each food or beverage item, consumption was calculated by multiplying the reported number of servings by a standard portion size. Built-in algorithms analyzed food composition and calculated specific nutrient intakes, including total sugar intake [15], [16], [17]. The validity of the Oxford WebQ has been demonstrated through comparisons with traditional interviewer-led 24-hour dietary recalls, particularly when multiple questionnaires are completed [18].

In this study, data on total sugar intake (g/day) and sugar subtypes (free sugar, fructose, glucose, sucrose, maltose, lactose, and other sugars) were extracted and analyzed. Participants were categorized into tertiles based on their daily intake of each sugar type, forming three groups: Q1 (lowest intake), Q2, and Q3 (highest intake), with Q1 serving as the reference group for analysis.

### 2.3 Outcome ascertainment

The primary outcome variable for this study, referred to as all-cause dementia (hereafter “dementia”), was defined by an algorithm provided by the UK Biobank. This algorithm determined the date of dementia diagnosis based on linked data from hospital admission records, death registries, and self-reported medical conditions at baseline. Additionally, first-occurrence data from primary care visits were included to complement the algorithm-defined dementia outcomes. To enhance the accuracy of dementia diagnosis, individuals diagnosed with dementia at baseline were excluded. Follow-up time was defined as the period from the first dietary assessment to the earliest of the following events: the first dementia diagnosis, loss to follow-up, death, or the end of the study review [19,20]. Dementia diagnoses were made using the International Classification of Diseases (ICD-10) codes F00-F03, G30, G310, G311, and G318.

### 2.4 Covariates

Covariates were selected based on previously established risk factors for dementia and included sex (female/male), baseline age, race (White/non-White, including mixed race, Black, Asian, and other ethnic groups), body mass index (BMI), Townsend Deprivation Index (TDI), education level (Degree level or professional education, Other levels, and Missing value), smoking status, alcohol consumption, use of vitamin or mineral supplements, physical activity level (assessed using the International Physical Activity Questionnaire [IPAQ] and categorized as low, medium, or high), and medical conditions including diabetes, hypertension, and obesity [21].

### 2.5 Data analysis

Baseline characteristics were summarized according to tertiles of total sugar intake. Continuous variables were presented as means with standard deviations (SD), while categorical variables were reported as counts and percentages.

A Cox proportional hazards regression model was employed to assess the association between sugar intake and dementia risk. Sugar intake was categorized into tertiles (Q1 to Q3), with Q1 serving as the reference group. Covariates were adjusted in three models: Model 1 included sociodemographic factors (age, sex, ethnicity, education level, and TDI); Model 2 additionally incorporated behavioral factors, including BMI, smoking status, alcohol consumption, IPAQ score, and supplement use; and Model 3 further adjusted for medical conditions, including diabetes, cancer, hypertension, and cardiovascular disease. The proportional hazards (PH) assumption was thoroughly evaluated for all Cox regression models included in our study. Specifically, we assessed this assumption using Schoenfeld residuals via the cox.zph function in R.

Hazard ratios (HRs) and 95 % confidence intervals (CIs) were calculated, and p-values for trend were computed using the median value of each tertile as a quasi-continuous variable in the model. Stratified analyses were conducted by sex to explore potential sex-specific associations. To further assess whether sex modifies the association between sugar intake and dementia risk, we included interaction terms between sex and sugar intake variables in the Cox proportional hazards models. We tested the significance and consistency of these interactions across different model specifications and subsamples to evaluate the potential effect modification by sex and the robustness of the interaction effects.

All statistical analyses were performed using the R programming language, with a significance threshold set at *p* < 0.05. Given that ten independent tests were conducted, Bonferroni correction was applied, resulting in an adjusted significance level of 0.005 (0.05 / 10). Only results with *p*-values < 0.005 were considered statistically significant.

## 3 Results

### 3.1 Baseline characteristics of UK Biobank participants

Based on tertiles of total sugar intake (Q1 to Q3, representing progressively higher intake), the characteristics of the study population are summarized in Table 1. The number of participants in the Q1, Q2, and Q3 groups was 57,506, 57,505, and 57,505, respectively. Notably, the mean age of participants increased across tertiles: 55.0 ± 8.0 years in Q1, 56.1 ± 7.9 years in Q2, and 56.6 ± 7.9 years in Q3, suggesting a trend of higher mean age with increasing sugar intake. Regarding sex distribution, females were fewer than males in the Q1 group, whereas females outnumbered males in both the Q2 and Q3 groups.

**Table 1:** Baseline characteristics of the study population.

Characteristics	Total sugar intake		
	Q1	Q2	Q3
Number	57,506	57,505	57,505
Age	55.0 (8.0)	56.1 (7.9)	56.6(7.9)
Sex			
Female	27,990 (48.7)	32,294 (56.2)	32,130 (55.9)
Male	29,516 (51.3)	25,211 (43.8)	25,375 (44.1)
Racial			
white	55,298 (96.2)	55,443 (96.4)	54,750 (95.2)
Non-white	2208 (3.8)	2062 (3.6)	2755 (4.8)
TDI	−1.50 (2.9)	−1.70 (2.8)	−1.63 (2.9)
BMI	27.28 (4.7)	26.65 (4.5)	26.65 (4.5)
IPAQ			
Low	12,208 (21.2)	10,369 (18.0)	8996 (15.6)
Moderate	24,816 (43.2)	24,947 (43.4)	23,458 (40.8)
High	20,482 (35.6)	22,189 (38.6)	25,051 (43.6)
Education level			
Degree/Professional	31,538 (54.8)	32,070 (55.8)	31,058 (54.0)
Other	21,824 (38.0)	21,257 (37.0)	21,763 (37.8)
Missing value	4144 (7.2)	4178 (7.3)	4684 (8.1)
Alcohol intake, Yes	56,493 (98.2)	55,947 (97.3)	54,861 (95.4)
Smoking, Yes	27,685 (48.1)	24,280 (42.2)	23,013 (40.0)
Supplementation, Yes	24,584 (42.8)	29,014 (50.5)	31,471 (54.7)
Medical history			
Hypertension, Yes	15,633 (27.2)	15,093 (26.2)	15,920 (27.7)
CVD,Yes	10,042 (17.5)	9857 (17.1)	10,708 (18.6)
Cancer, Yes	4945 (8.6)	5414 (9.4)	5657 (9.8)
Diabetes, Yes	4654 (8.1)	3485 (6.1)	3330 (5.8)

Notes: Data is presented as mean (SD) for continuous variables and as frequency (percentage) for categorical variables. BMI, body mass index; TDI, Townsend Deprivation Index; IPAQ, International Physical Activity Questionnaire.

### 3.2 Sugar intake and dementia

The PH assumption showed *p*-values > 0.05, and the residual plots demonstrated approximately flat trend lines over time, providing further support for the validity of the PH assumption. The detailed results of the PH assumption tests are provided in Supplementary Table 1.

In a series of analyses examining the effects of total and free sugar intake on dementia incidence, three models (Model 1, Model 2, and Model 3) were constructed for in-depth evaluation. The results indicated that the highest intake levels of both total and free sugars were significantly associated with increased risk of dementia. Specifically, the hazard ratio (HR) and 95 % confidence interval (CI) for the highest total sugar intake group were 1.292 (1.148, 1.453). Similarly, the highest free sugar intake group had an HR of 1.254 (1.117, 1.408). Moreover, the highest intake groups of non-milk extrinsic sugars and sucrose were also significantly associated with elevated dementia risk, with HRs (95 % CIs) of 1.321 (1.175, 1.486) and 1.291 (1.147, 1.452), respectively. In this study, only the results from Model 3 are presented in Table 2, as they best represent the final specification and key findings of the analysis.

**Table 2:** Associations between sugar intake and dementia.

Sugar	Quartile	Model 1		Model 2		Model 3	
		HR (95 %CI)	*P*-Value	HR (95 %CI)	*P*-Value	HR (95 %CI)	*P*-Value
Total sugars	Q1	Ref					
	Q2	0.953 (0.840, 1.080)	4.49 × 10^–1^	0.965(0.850, 1.095)	5.77 × 10^–1^	0.980(0.863, 1.112)	7.50 × 10^–1^
	Q3	1.266(1.127, 1.422)	6.85 × 10^–5^	1.291(1.148, 1.452)	2.08 × 10^–5^	1.292(1.148, 1.453)	2.02 × 10^–5^
Free sugar	Q2	1.033(0.916, 1.165)	5.99 × 10^–1^	1.037(0.919, 1.170)	5.56 × 10^–1^	1.073(0.951, 1.211)	2.50 × 10^–1^
	Q3	1.224(1.091, 1.374)	5.90 × 10^–4^	1.228(1.094, 1.379)	4.96 × 10^–4^	1.254(1.117, 1.408)	1.31 × 10^–4^
Fructose	Q2	0.865 (0.764, 0.979)	2.19 × 10^–2^	0.875(0.772, 0.991)	3.57 × 10^–2^	0.879(0.776, 0.995)	4.22 × 10^–2^
	Q3	1.115(0.993, 1.252)	6.49 × 10^–2^	1.138(1.012, 1.280)	3.06 × 10^–2^	1.150(1.023, 1.294)	1.97 × 10^–2^
Glucose	Q2	0.936(0.827, 1.059)	2.94 × 10^–1^	0.946(0.835, 1.070)	3.76 × 10^–1^	0.955(0.844, 1.081)	4.70 × 10^–1^
	Q3	1.133(1.009, 1.273)	3.50 × 10^–2^	1.155(1.026, 1.299)	1.67 × 10^–2^	1.167(1.037, 1.313)	1.04 × 10^–2^
Lactose	Q2	0.901(0.797, 1.019)	9.56 × 10^–2^	0.907(0.803, 1.026)	1.21 × 10^–1^	0.920(0.814, 1.040)	1.82 × 10^–1^
	Q3	1.101(0.981, 1.235)	1.03 × 10^–1^	1.114(0.993, 1.251)	6.65 × 10^–2^	1.117(0.995, 1.253)	6.14 × 10^–2^
Maltose	Q2	0.973(0.866, 1.093)	6.44 × 10^–1^	0.974(0.866, 1.095)	6.56 × 10^–1^	0.967(0.861, 1.088)	5.79 × 10^–1^
	Q3	1.000(0.889, 1.125)	9.96 × 10^–1^	0.995(0.884, 1.119)	9.30 × 10^–1^	0.982(0.873, 1.104)	7.60 × 10^–1^
Intrinsic and milk sugars	Q2	0.969(0.855, 1.098)	6.20 × 10^–1^	0.982(0.867, 1.113)	7.79 × 10^–1^	0.986(0.870, 1.118)	8.24 × 10^–1^
	Q3	1.145(1.017, 1.290)	2.55 × 10^–2^	1.170(1.037, 1.320)	1.06 × 10^–2^	1.157(1.025, 1.305)	1.81 × 10^–2^
Non-milk extrinsic sugars	Q2	1.086(0.961,1.226)	1.84 × 10^–1^	1.094(0.968, 1.1236)	1.49 × 10^–1^	1.132(1.002, 1.279)	4.65 × 10^–2^
	Q3	1.285(1.144,1.444)	2.30 × 10^–5^	1.294(1.151, 1.454)	1.50 × 10^–5^	1.321(1.175, 1.486)	3.11 × 10^–6^
Sucrose	Q2	1.088(0.962, 1.230)	1.79 × 10^–1^	1.099(0.972, 1.243)	1.31 × 10^–1^	1.108(0.980, 1.254)	1.02 × 10^–1^
	Q3	1.278(1.137, 1.436)	4.05 × 10^–5^	1.290(1.147, 1.451)	2.18 × 10^–5^	1.291(1.147, 1.452)	2.21 × 10^–5^
Other Sugars	Q2	1.024(0.908, 1.154)	7.04 × 10^–1^	1.028(0.912, 1.160)	6.50 × 10^–1^	1.051(0.931, 1.185)	4.21 × 10^–1^
	Q3	1.189(1.057, 1.337)	3.95 × 10^–3^	1.190(1.058, 1.339)	3.75 × 10^–3^	1.180(1.049, 1.328)	5.83 × 10^–3^

Notes:

Model 1: Adjusted for sociodemographic factors, including age, sex, ethnicity, education level, and TDI.

Model 2: Additionally adjusted for behavioral factors, including BMI, smoking status, alcohol consumption, IPAQ, and supplement use.

Model 3: Further adjusted for medical conditions, including diabetes, cancer, hypertension, and cardiovascular disease.

Abbreviations: HR, hazards ratio; CI, confidence interval; Ref, reference; Q, quartile.

### 3.3 Stratified analyses

**Fig. 1:**
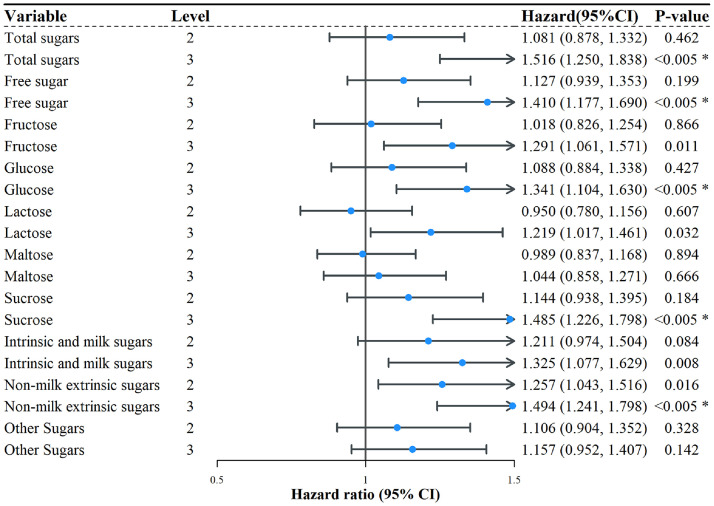
Association between sugar intake and the risk of dementia in the female subgroup Notes: Blue dots indicate the hazard ratio (HR), and the connecting line represents the 95 % confidence interval (CI).

**Fig. 2:**
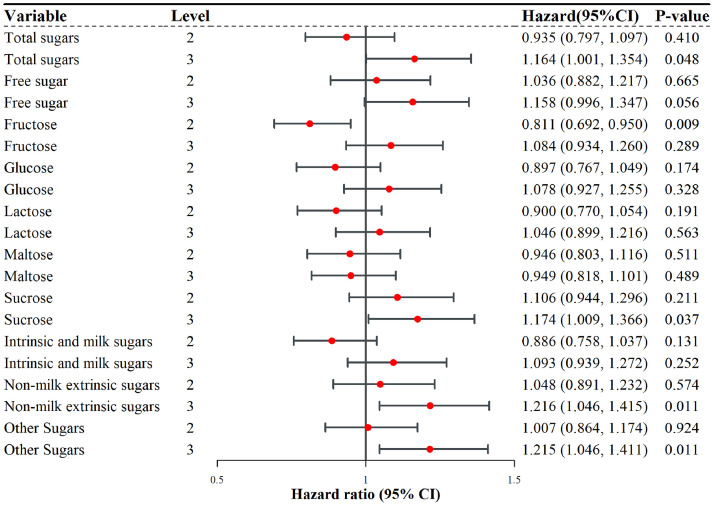
Association between sugar intake and the risk of dementia in the male subgroup Notes: Red dots indicate the hazard ratio (HR), and the connecting line represents the 95 % confidence interval (CI).

We stratified the population by sex and observed significant associations between sugar intake and dementia risk among females. Specifically, higher intakes of total sugars (HR = 1.516, 95 % CI: 1.250–1.838), free sugars (HR = 1.410, 95 % CI: 1.177–1.690), glucose (HR = 1.341, 95 % CI: 1.104–1.630), sucrose (HR = 1.485, 95 % CI: 1.226–1.798), and non-milk extrinsic sugars (HR = 1.494, 95 % CI: 1.241–1.798) were significantly associated with increased dementia risk. In contrast, no significant associations were found between total or subtype-specific sugar intake and dementia risk in males. However, after incorporating the sugar × sex interaction terms into the Cox models, the interaction effects were not statistically significant, indicating no meaningful difference in the associations between sexes. These findings are presented in Fig. 1, Fig. 2, while the interaction results are provided in Fig. S1.

## 4 Discussion

This study examined the association between sugar intake and dementia risk using data from a large UK Biobank cohort. Higher consumption of total sugars, free sugars, non-milk extrinsic sugars, and sucrose was independently associated with an increased risk of dementia. Stratified analyses revealed significant associations between dementia risk and the intake of total sugars, free sugars, glucose, sucrose, and non-milk extrinsic sugars, particularly among females. These results align with previous research and support the hypothesis that a high-sugar diet constitutes a significant risk factor for dementia [22].

To our knowledge, this is the first prospective cohort study in a general population to systematically investigate the dose-response relationship between total sugar intake, its subtypes—including free sugars, fructose, glucose, sucrose, lactose, and other sugars—and incident dementia. Current evidence on dietary sugars and dementia risk remains limited and primarily focuses on composite dietary patterns. For example, Zhang et al. reported a positive association between high-sugar dietary patterns and dementia risk in a prospective cohort study, but their analysis did not differentiate among sugar subtypes, leaving unclear whether specific sugars (e.g., free sugars, fructose, glucose, sucrose, maltose, milk extrinsic sugars) contribute differently to dementia risk [23]. In contrast, our study not only reaffirmed the significant association between high sugar intake and dementia risk but also made an important analytical advance by carefully distinguishing sugar subtypes—including free sugars, fructose, and sucrose—and examining their distinct effects on dementia risk. This novel approach overcomes previous limitations and establishes a robust foundation for further mechanistic research.

Although a significant association between sugar intake and dementia risk was observed, the underlying mechanisms remain incompletely understood. Existing studies suggest several pathways through which a high-sugar diet may increase dementia risk, including insulin resistance, oxidative stress, inflammatory responses, and alterations in the gut microbiome [24], [25], [26], [27]. Insulin resistance, a hallmark of metabolic dysfunction commonly associated with high sugar consumption, has been increasingly implicated in the development and progression of dementia. Chronic consumption of high-sugar diets may lead to systemic insulin resistance, which not only impairs peripheral metabolic regulation but also disrupts insulin signaling in the brain. This disruption leads to synaptic dysfunction, reduced neuronal plasticity, and ultimately, cognitive decline. Neuronal insulin signaling plays a critical role in maintaining cognitive function, and its impairment may hinder amyloid-β (Aβ) clearance, promote abnormal tau phosphorylation, and induce synaptic damage—hallmarks of Alzheimer’s disease and related dementias. Although the present study did not directly examine these biological pathways, the observed associations at the population level are consistent with existing mechanistic research. These findings provide indirect support for the role of metabolic dysregulation in dementia and contribute additional epidemiological evidence regarding the potential impact of dietary sugar intake on cognitive health [28,29]. Furthermore, high sugar consumption may exacerbate neuronal damage by increasing oxidative stress. Oxidative stress contributes to mitochondrial dysfunction, lipid peroxidation, and neuronal apoptosis, all of which are central features of neurodegeneration. It may also damage the cerebral vascular endothelium, disrupt the blood–brain barrier, and initiate neuroinflammatory responses, thereby accelerating cognitive decline [30,31]. A high-sugar diet may also alter gut microbiota composition by increasing harmful bacteria, which can affect brain function via systemic circulation and exacerbate neuropathological changes [32,33]. Despite these insights, further experimental and mechanistic studies are needed to fully elucidate how sugar intake influences dementia risk.

Furthermore, our stratified analyses revealed a sex-specific association between sugar intake and dementia risk. Previous research shows that women differ from men in neuroendocrine regulation, metabolic profiles, and behavior, potentially increasing their vulnerability to impaired glucose metabolism and neurodegenerative diseases [34,35]. For instance, estrogen decline after menopause may exacerbate the harmful effects of a high-sugar diet on hippocampal glucose metabolism by reducing insulin sensitivity [36]. Metabolomics studies in Alzheimer’s disease (AD) mouse models have found that female mice display more severe impairments in glucose clearance, whereas male mice sometimes show improved clearance at certain ages [37]. Epidemiological data also indicate that women generally consume more sugar daily than men, and chronic sugar exposure may promote abnormal tau protein phosphorylation through the advanced glycation end products–receptor for advanced glycation end products (AGEs–RAGE) pathway, aggravating neurodegeneration [38,39]. These sex-related biological differences provide valuable insights into how sugar intake influences dementia risk, underscoring sex as a critical factor in glucose metabolism and dementia development. Further analysis of the sex × sugar intake interaction did not reach statistical significance, indicating that although stratified analyses revealed significant effects in females, the moderating role of sex in this association remains statistically inconclusive. This outcome may be due to the stringent significance threshold (*p* < 0.005) employed in this study to mitigate the risk of false positives arising from multiple testing. Although some stratified analyses showed significant associations, the interaction test did not meet this strict criterion, suggesting that sex differences in the effect warrant confirmation through larger sample sizes or independent cohorts.

Despite these important findings, several limitations warrant consideration and suggest directions for future research. First, dietary assessment limitations must be acknowledged. Although combining the Oxford WebQ and 24-hour dietary recalls improved data reliability, sugar intake was ultimately self-reported, which is subject to systematic underreporting of high-sugar foods due to social desirability bias [40]. Despite data adjustments (e.g., portion size correction, frequency conversion, nutrient algorithm calculations), changes in dietary habits during follow-up (e.g., voluntary sugar reduction) may not have been fully captured. Future studies integrating objective biomarkers, such as urinary glucose metabolites, could enhance exposure measurement accuracy. Second, due to the non-random availability of dietary recall data in the UK Biobank, concerns about the generalizability of the study findings arise. Participants who completed the dietary assessments may differ systematically from those who did not, potentially introducing selection bias. Additionally, a “healthy volunteer” selection bias within the UK Biobank cohort may have contributed to an underestimation of the observed positive associations, as participants tend to be healthier than nonparticipants [41]. Moreover, the majority of participants in the UK Biobank were White British, which may limit the generalizability of our findings to more ethnically diverse populations [23]. Third, this study did not directly investigate key mechanistic mediators linking sugar intake to dementia. Although sugar subtypes were distinguished for the first time, crucial molecular markers like advanced glycation end products (AGEs) were not measured [42,43], limiting subtype-specific neurotoxicity analysis. The absence of insulin resistance indices (e.g., HOMA-IR) and systemic inflammatory markers (e.g., IL-6, CRP) also precluded a comprehensive multi-omics framework [44]. Fourth, although participants with baseline dementia were excluded, individuals with other neurological disorders, such as stroke and Parkinson’s disease, were neither excluded nor adjusted for in the analyses, which may have introduced residual confounding. This represents a limitation of our study. Future research should consider addressing this issue to better clarify the relationship between exposure and dementia risk. Fifth, to ensure data quality, participants with physiologically implausible energy intakes—defined as less than 500 or more than 3500 kcal/day for women, and less than 800 or more than 4000 kcal/day for men—were excluded based on established criteria. Extremely low or high reported energy intakes often reflect reporting errors, recall bias, or technical issues during data entry. Including such implausible dietary records can distort dose–response relationships and increase measurement error, thereby reducing the statistical power of the analysis. Future studies using objective biomarkers of dietary intake could help overcome these limitations.

## 5 Conclusions

The present study confirmed a significant association between a high-sugar diet and dementia risk through prospective cohort analysis, revealing notable sex differences. Specifically, the association was more pronounced among women. These findings underscore the importance of developing targeted dietary intervention strategies and suggest that public health policies should prioritize stricter restrictions on high-sugar diets, particularly for the female population. Future research should further investigate the biological mechanisms underlying the relationship between sugar intake and dementia risk, incorporating multi-omics approaches and neuroimaging data to establish a more comprehensive scientific foundation for dementia prevention and treatment.

## Statement of ethics

Ethical approval of the UKB study was granted by the National Health Service National Research Ethics Service (reference 11/NW/0382).

## Funding

The Natural Science Basic Research Plan in Shaanxi Province of China [2021JCW-08].

## CRediT authorship contribution statement

**Yue Che:** Writing – original draft, Methodology, Conceptualization. **Wenming Wei:** Writing – original draft, Visualization. **Tingting Mao:** Visualization. **Lina Qin:** Writing – original draft. **Hanchi Wang:** Methodology. **Yijia Li:** Methodology. **Weixuan Da:** Conceptualization. **Jin Feng:** Supervision. **Li Liu:** Methodology, Conceptualization. **Bolun Cheng:** Methodology, Formal analysis. **Huan Liu:** Supervision, Data curation. **Yan Wen:** Supervision. **Yumeng Jia:** Supervision, Data curation. **Feng Zhang:** Writing – review & editing, Methodology, Funding acquisition.

## Declaration of competing interest

The authors have stated that they have no conflict of interest.

## Data Availability

The data supporting the findings of this study are available from the UK Biobank. Access to the UK Biobank resource is available to qualified researchers through an application process via their official website (http://www.ukbiobank.ac.uk/about-biobank-uk/).

## Appendix Supplementary materials

Image, application 1
