# Supplementary material for: Association of sugar intake with incident dementia in the UK Biobank: a prospective cohort study
Source: J Prev Alzheimers Dis. 2025 Aug 5;12(9):100311. doi: 10.1016/j.tjpad.2025.100311 (PMC12501338; doi:10.1016/j.tjpad.2025.100311)
Supplement: Supplementary file 1 [file mmc1.docx]

**Legends for Tables and Figures**

**Table S1：**Test of Proportional Hazards Assumption for Sugar Intake Models Using Schoenfeld Residuals

**Figure S1:** Interaction Effect of Sex on the Association Between Sugar Intake and Dementia Risk

Notes: Green dots indicate the hazard ratio (HR), and the connecting line represents the 95% confidence interval (CI).

Table S1. Test of Proportional Hazards Assumption for Sugar Intake Models Using Schoenfeld Residuals

| Sugars | Model | Exposure *p*-value | Global *p*-value |
| --- | --- | --- | --- |
| Total sugars | Model1 | 6.71×10^-1^ | 5.25×10^-1^ |
|  | Model2 | 6.61×10^-1^ | 9.33×10^-2^ |
|  | Model3 | 6.97×10^-1^ | 2.72×10^-1^ |
| Free sugar | Model1 | 9.51×10^-1^ | 5.58×10^-1^ |
|  | Model2 | 9.50×10^-1^ | 9.81×10^-2^ |
|  | Model3 | 9.67×10^-1^ | 2.83×10^-1^ |
| Fructose | Model1 | 7.31×10^-1^ | 5.16×10^-1^ |
|  | Model2 | 7.23×10^-1^ | 9.41×10^-2^ |
|  | Model3 | 7.00×10^-1^ | 2.72×10^-1^ |
| Glucose | Model1 | 5.60×10^-1^ | 4.83×10^-1^ |
|  | Model2 | 5.48×10^-1^ | 8.90×10^-2^ |
|  | Model3 | 5.53×10^-1^ | 2.62×10^-1^ |
| Lactose | Model1 | 9.00×10^-1^ | 5.56×10^-1^ |
|  | Model2 | 8.89×10^-1^ | 9.74×10^-2^ |
|  | Model3 | 9.63×10^-1^ | 2.83×10^-1^ |
| Maltose | Model1 | 7.78×10^-1^ | 5.65×10^-1^ |
|  | Model2 | 7.81×10^-1^ | 9.79×10^-2^ |
|  | Model3 | 8.74×10^-1^ | 2.83×10^-1^ |
| Intrinsic and milk sugars | Model1 | 6.92×10^-1^ | 5.08×10^-1^ |
|  | Model2 | 6.87×10^-1^ | 9.09×10^-2^ |
|  | Model3 | 6.93×10^-1^ | 2.68×10^-1^ |
| Non-milk extrinsic sugars | Model1 | 3.96×10^-1^ | 4.91×10^-1^ |
|  | Model2 | 3.91×10^-1^ | 8.75×10^-2^ |
|  | Model3 | 4.19×10^-1^ | 2.61×10^-1^ |
| Sucrose | Model1 | 5.25×10^-1^ | 5.06×10^-1^ |
|  | Model2 | 5.22×10^-1^ | 8.94×10^-2^ |
|  | Model3 | 5.54×10^-1^ | 2.66×10^-1^ |
| Other Sugars | Model1 | 3.64×10^-1^ | 4.15×10^-1^ |
|  | Model2 | 3.67×10^-1^ | 7.47×10^-2^ |
|  | Model3 | 4.15×10^-1^ | 2.40×10^-1^ |

Notes: Exposure *p*-value: *p*-value for the Schoenfeld residual test of the exposure variable (sugar intake). Global *p*-value: *p*-value for the global Schoenfeld test across all covariates in the model.

Figure S1: Interaction Effect of Sex on the Association Between Sugar Intake and Dementia Risk


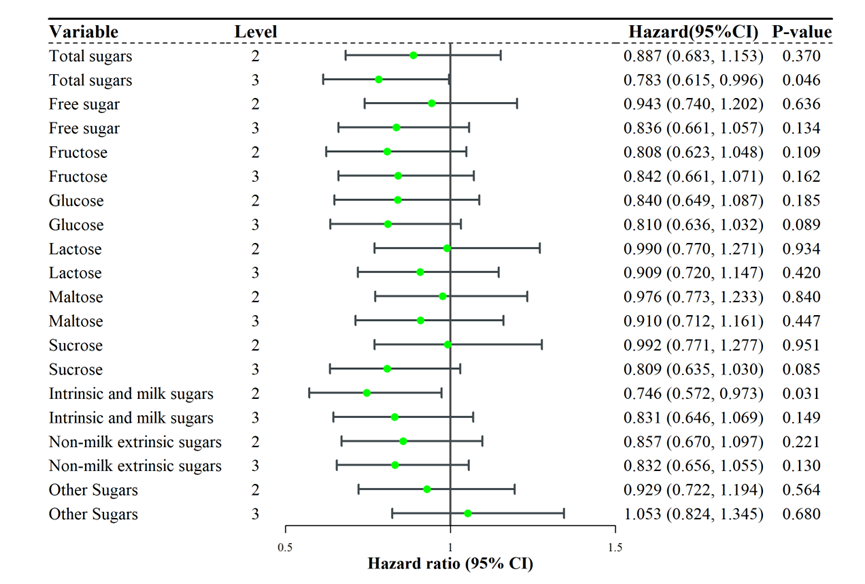


Notes: Green dots indicate the hazard ratio (HR), and the connecting line represents the 95% confidence interval (CI).
